# Angina Leading to Metal in the Heart: An Interesting Case of Saphenous Vein Graft Coiling

**DOI:** 10.7759/cureus.7546

**Published:** 2020-04-05

**Authors:** Raj Patel, Harshavardhan Ghadiam, Puja Patel, John Rashid

**Affiliations:** 1 Cardiology, University of Illinois College of Medicine at Peoria, Peoria, USA; 2 Internal Medicine, American University of Antigua, Brooklyn, USA; 3 Interventional Cardiology, University of Illinois College of Medicine at Peoria, Peoria, USA

**Keywords:** vein graft, interventional cardiology, cardiology devices, catheterization

## Abstract

This is an interesting coronary angiography and interventional cardiology case of a 75-year-old Caucasian male with a prior history of coronary artery bypass surgery who presented with non-ST elevation myocardial infarction (NSTEMI) thought to be secondary to distal embolization from thrombus in large right coronary artery (RCA) vein graft aneurysms. This subsequently resulted in percutaneous intervention with coiling of the aneurysmal vein graft segments.

## Introduction

Saphenous vein graft aneurysms (VGA) are rare, however when they do occur, they may result in major clinical complications, such as myocardial ischemia, vein graft rupture, compression of adjacent structures and even death. Mainstays of treatment for VGA include conservative management with long-term anti-coagulation and in severe cases, coiling of the aneurysmal dilitations. This case highlights the use of coils in the management and treatment of severe vein graft aneurysms [1].

## Case presentation

**Figure 1:**
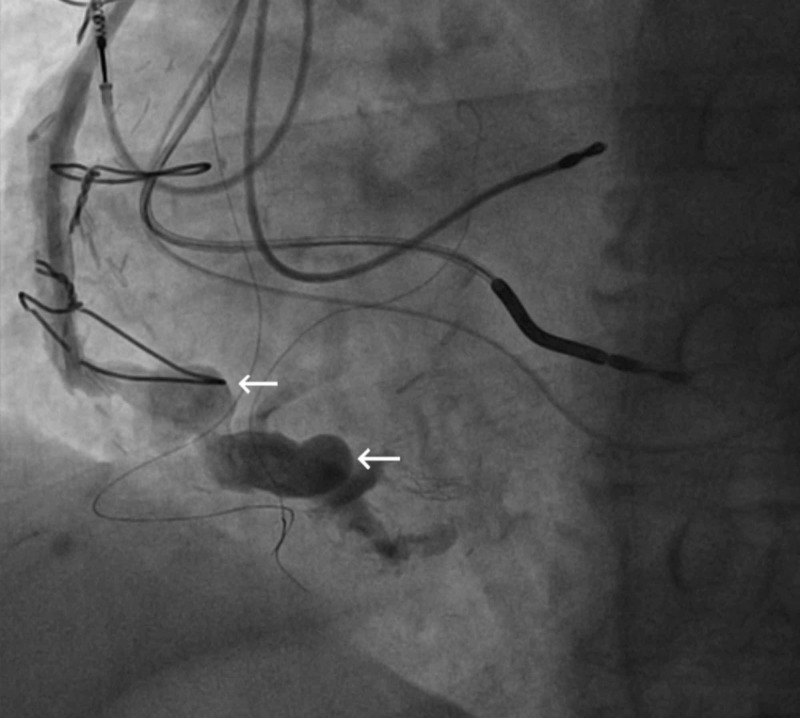
Diagnostic coronary angiogram revealing several right coronary artery vein graft aneurysms (see arrows).

**Figure 2:**
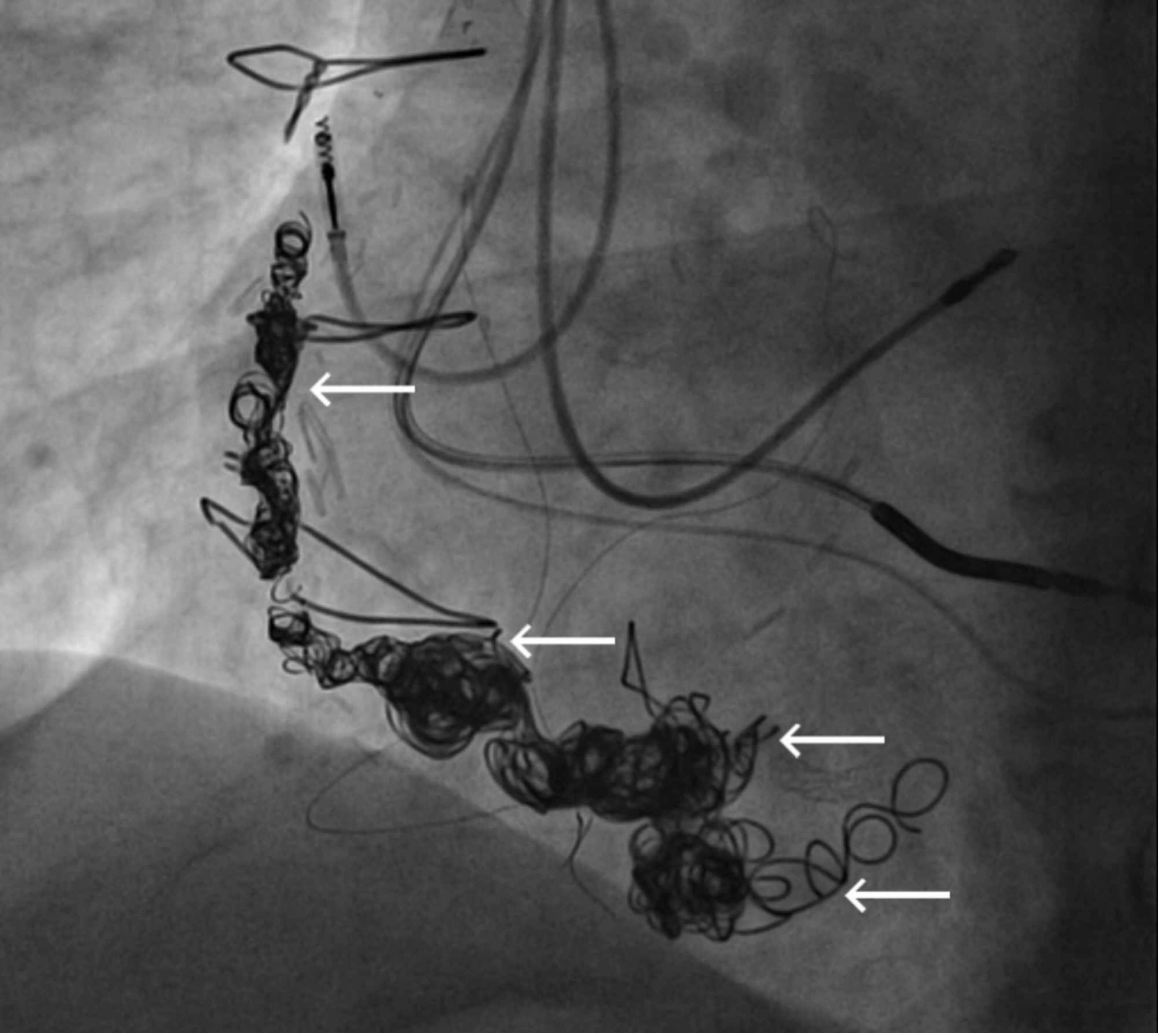
Post-coiling of right coronary artery saphenous vein graft aneurysms (see arrows).

**Figure 3:**
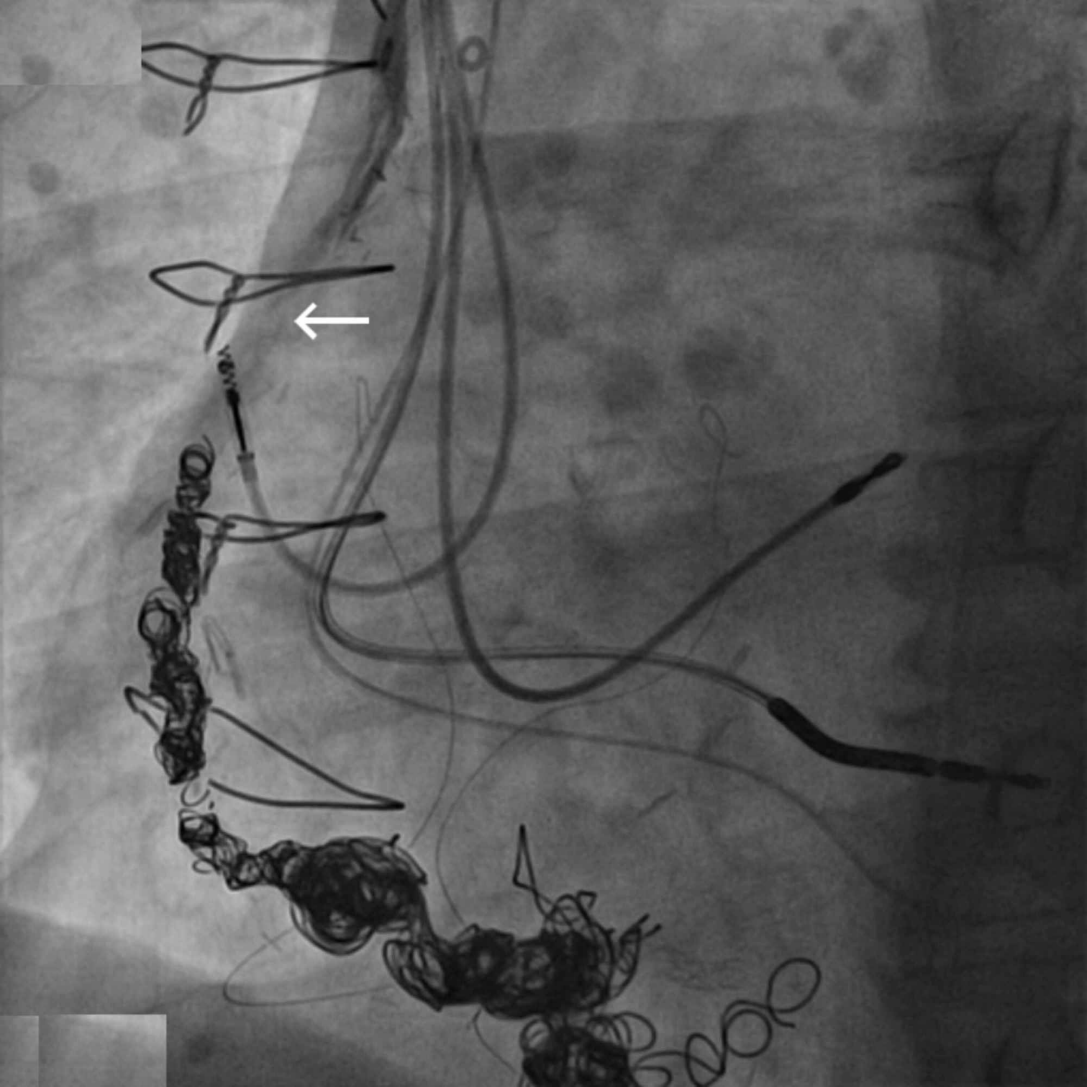
Saphenous vein graft aneurysm angiography post coiling with evidence of procedural success due to lack of distal contrast filling (see arrow).

We present the case of a 75-year-old Caucasian male with a past medical history of coronary artery disease s/p coronary artery bypass surgery in 1998 with subsequent development of right coronary artery (RCA) VGA, ischemic cardiomyopathy s/p implantable cardioverter defibrillator (ICD) implantation, essential hypertension and hyperlipidemia who presented to the hospital with non-ST elevation myocardial infarction (NSTEMI) in the spring of 2018. Prior to the NSTEMI, a chest computed tomography scan in 2017 done for respiratory issues revealed progression of a known RCA aneurysm. He was subsequently started on warfarin therapy. Due to progressively increasing size of the VGA, an outpatient coiling procedure was planned. The patient was taken off of chronic warfarin therapy in anticipation of outpatient VGA coiling, however he subsequently presented with an NSTEMI a few days prior to his planned procedure. He underwent cardiac catheterization and coronary angiography which did not reveal any significant or new luminal irregularities in the bypass grafts or native coronary arteries to explain the NSTEMI presentation. It was concluded that distal embolization from thrombus within the RCA VGA in the setting of cessation of anticoagulation (see Figure 1) was the likely culprit. Thus, an urgent procedure to occlude the aneurysmal dilations in the RCA VGA was planned. Using a 6F system, the mid-distal vein graft was engaged with a multipurpose guide catheter. Interventional radiology team assisted in the placement of 18 coils to occlude flow within the aneurysmal segments (see Figure 2). Cessation of flow was confirmed after the placement of coils (see Figure 3). The patient’s anticoagulation was resumed with a plan for lifelong therapy and he was discharged to home the following day in stable condition.

## Discussion

Aneurysms are generally defined as a local enlargement involving all three vessel walls and usually results in a vessel size 1.5 times greater than the proximal reference diameter. Vein graft aneurysms can be classified into different sub-groups based on timing of aneurysm formation in reference to index coronary artery bypass grafting (CABG) procedure. Early aneurysm formation is defined <12 months after surgery. Late aneurysm formation is defined as >5 years post surgery [1].

It should be noted that pseudo-aneurysms can also occur and can be mistaken for true aneurysms. The definition of a pseudo-aneurysm is an out-pouching resulting from one or two layers of the vessel wall being dilated [2]. Generally, the aneurysm develops a hematoma and usually involves the proximal and distal ends of the vein graft [3]. Pseudo-aneurysms are generally more common than true aneurysms [1].

Treatment of true saphenous vein graft aneurysms is undertaken to prevent complications of the pathology. Rupture, myocardial ischemia, and adjacent mass effect are common manifestations of saphenous vein graft aneurysms [3]. Several percutaneous interventions have been developed to decrease the risk of adverse outcomes from aneurysms. Vascular plugs, coil embolization, covered stunting and alcohol injection into the graft are some techniques used to treat the dilations [1].

Additionally, clinicians should consider medical therapy either in place of or in conjunction with percutaneous therapies. Aspirin concurrently with warfarin or novel oral anticoagulant therapy can be considered to reduce the risk of vein graft thrombus formation [3].

## Conclusions

Saphenous vein graft aneurysms, although rare, have dramatic clinical manifestations and have several treatment modalities including medical and interventional therapies. This case represents the use of coiling to treat aneurysmal dilation of an RCA vein graft and highlights the advantage of percutaneous procedures in this subset of patients.
